# The Effects of Environmental Tax Revenue on Sustainable Development in China

**DOI:** 10.3390/ijerph20065022

**Published:** 2023-03-12

**Authors:** Bingjie Wang, Chong Xu, Ding Li, Yinyin Wu, Yaqi Zhang

**Affiliations:** 1 School of Public Finance and Taxation, Southwestern University of Finance and Economics, Chengdu 610074, China; 2 School of Public Administration, Southwestern University of Finance and Economics, Chengdu 610074, China; 3 Business School, The University of Hong Kong, Hong Kong, China

**Keywords:** environmental tax revenue, sustainable development, China

## Abstract

Despite extensive studies focused on environmental tax revenue (ETR) on the driver and linkage with socioeconomic variables over time, an in-depth investigation on the spatiotemporal driver and intrinsic characteristics (e.g., convergence and complex network) is in need, providing valuable information on formulating better environmental tax policy towards sustainable development. Therefore, the study comprehensively analyzed the spatiotemporal driver, convergence trend, and complex network of provincial ETR in a case of China over 2000–2019 by using temporal and spatial logarithmic mean Divisia index models (LMDI), convergence models, and social network analysis, respectively. We found that, first, two convergence clubs of ETR for China’s provinces over the period were found. Second, GDP per capita and tax intensity were the positive and negative drivers contributing the increase in ETR. Third, within differences in tax intensity and GDP per capita, as well as the differences in population and GDP per capita, were the main drivers widening the overall ETR gap. Fourth, the original hierarchical ETR spatial correlation structure has changed, while provinces exhibited certain degrees of heterogeneity in terms of ETR spatial association network. The study highlights that ETR plays a significant role in maintaining sustainable development and thus suggests that more importance of environmental tax policies at various levels should be attached.

## 1. Introduction

In many early industrialized countries, rapid economic growth is often accompanied by increasingly serious air pollution problems, such as early London in the United Kingdom and the Ruhr Industrial Base in Germany. Industrialization and urbanization are still in progress for many emerging economies, which seems to inevitably contribute to the pollution problem. Unlike consumption tax and corporate tax [1,2,3], environmental tax is widely used to control environmental pollution [4] as one of the market-based tools. However, there are some differences in the contents of environmental tax due to the different patterns of economic development in different countries. Although environmental tax includes several taxes, such as emission tax and energy tax, those taxes essentially reflect the concept of sustainable development [5,6,7].

With the context above, the existing studies have analyzed environmental tax from various perspectives, with focuses on the driver and linkage with other socioeconomic variables. For example, Castiglione et al. (2018) showed that the determinants of ETR were different for different EU countries due to the institution and economic development by comparing three indices of environmental tax [8]. Further, Andreoni (2019) decomposed the change in ETR into three drivers by LMDI in EU countries and found that economic growth contributed to the increase in ETR [5]. After discussing the impact of environmental tax on green economic development by utilizing dynamical system approach, Fan et al. (2019) argued that environmental tax benefits developed green economic development [9]. Bashir et al. (2021) found the positive role of environmental tax in reducing energy usage and energy intensity in selected countries [10]. A study analyzed the different ratio of ETR refunded to consumption tax on the national economy in China by the Computable General Equilibrium (CGE) model and showed that environmental tax can curb high pollution without hurting the economy [11]. Further, Shang et al. (2022) found that environmental tax can stimulate the innovation motivation of enterprises in China [12].

Despite extensive studies focused on ETR from different perspectives above, the research on ETR is far from conclusive, and an in-depth investigation on ETR in a developing country is still in need in terms of spatiotemporal driver, convergence trend, and complex network, which has been documented by few researchers. Such deficiency may hinder the formulation of better environmental tax reforms towards sustainable development. The importance of multidimensional characteristics of ETR lies in the regional coordinated development, including economy and environment, which is supported by sustainable development theory [13]. The aspects analyzed were based on the index number theory for spatiotemporal drivers [14], economic equilibrium theory for convergence analysis [15], and complex network theory for network characteristics [16]. This is particularly true for a country such as China, the largest developing country with pollution problems. Moreover, unlike developed countries, China did not set up environmental taxes until 2018, and these were mainly based on pollution charge fees prior to that year, where ETRs are fees levied on pollutant discharge. Essentially, there is a high degree of connection and a certain difference between pollution discharge fee and environmental tax.

To be specific, first, the collection scope of environmental tax and the environmental pollution discharge fee are basically the same as they are for charges for the four categories of pollutants, including air, water, solid, and noise. However, the former only includes industrial noise, while the latter also includes construction noise and volatile organic compounds, additionally. Second, the standard of pollutant discharge fee is the same as that of enterprises, while the cost of environmental tax is dynamically adjusted according to the amount of pollutants. Third, there is one level for pollutant discharge fees and tow levels for environmental taxes in terms of emission reduction preferential treatment. Fourth, the environmental tax is collected and managed by the tax authority, and the environmental protection tax income is owned by the local government. Nevertheless, considering the impact of environmental tax on the economy, environmental protection taxes and pollution discharge fees present a high degree of consistency for the early stage of environmental tax, particularly.

Consequently, with the context above, the study attempted to comprehensively uncover the spatiotemporal driver, convergence, and complex network of ETR in a case of 30 provinces in mainland China during 2000–2019 by employing the spatiotemporal LMDI, the newly developed spatial within and between the LMDI approach, the convergence model, and the social network analysis (SNA), respectively, which have also been used in environment-related studies [17,18,19]. The study contributed the current literature, as follows.

First, unlike most previous studies focusing on driver of ETR over time [10,11], the study comprehensively investigated the spatiotemporal drivers of ETR by using temporal LMDI and newly developed spatials within and between LMDI approaches, simultaneously. Moreover, we conducted the study in the context of the largest developing country, China, which is different from most existing studies on the developed economy (see previous studies, [5,8], for example) and thus provides valuable insights on sustainable development for countries in transition, such as China in particular. Second, the study explored the long-term trend and complex network of ETR based on the newly developed convergence test and social network analysis, which have rarely been documented in the current literature (e.g., Andreoni (2019) and Castiglione et al. (2018)) [5,8]. Such new perspectives of ETR from convergence and complex networks can provide important references for policy makers and help to formulate policy implications towards better sustainable development based on environmental tax.

## 2. Materials and Methods

### 2.1. Convergence Model

The log t convergence test has the advantage of capturing the potential heterogeneity among regions [17,19], which is particularly true for the study due to the objective differences between provinces regarding ETR. Consequently, following the studies [15,20], let *X_it_* represent ETR in province *i* and year *t* (*i* = 1, 2,…, *N*; *t* = 1, 2, …, *T*). Then, we decompsed *X_it_* as:

(1) $$X_{it}=\delta_{it}\mu_{t}$$

where *µ_t_* means the common influencing factors of all provinces; and *δ_it_* is the ratio of common influencing factors for assessing provinces implying the transition path. *δ_it_* = *δ_i_* + *υ_i_ζ_it_L*(*t*)*t*^−*α*^), in which *δ_it_* represents a fixed effect, *υ_i_* > 0, while *ζ_it_* implies a standardized normal treatment for all provinces. *L*(*t*) means a function being set to a logarithmic form in accordance with Phillips and Sul (2007) [15].

We then used the following regression model to test convergence in accordance with the literature:

(2) $$\log\left(H_{1}/H_{t}\right)-2\log\left(\log t\right)=a+b\log\left(t\right)+\varepsilon_{t}$$

where t=[rT],[rT]+1,⋯,T, in which *r* is a portion of the initial sample discarded, which should be [0.2, 0.3] (Phillips and Sul, 2007) [15]; *H_t_* represents the variance of elative transition parameter [15], and $H_{t}=(1/N)\sum_{i=1}^{N}{\left(\delta_{it}/[1/N\sum_{i=1}^{N}\delta_{it}]-1\right)}^{2}$. Theoretically, the estimated coefficient in Equation (2) converges in probability under the original assumption, implying that $\hat{b}\overset{P}{\to }2\alpha \geq 0$. In accordance with Phillips and Sul (2007) [15], we then analyze the club convergence to reflect the regional heterogeneity.

### 2.2. General Temporal and Spatial LMDI Models

According to the Kaya identity theory (1989) [21], ETR for province *i* at year *t* can be defined as:

(3) $$ETR^{t}=\sum_{i}\frac{ETR_{i}^{t}}{Y_{i}^{t}}\times \frac{Y_{i}^{t}}{P_{i}^{t}}\times P_{i}^{t}=\sum_{i}TI_{i}^{t}\times PY_{i}^{t}\times P_{i}^{t}$$

Here, $ETR_{i}^{t}$ denotes ETR of the ith province at year t, $Y_{i}^{t}$ represents the GDP of the ith province at year t, and $Y^{t}$ represents the sum of sample provinces’ GDP at year t. Therefore, TI (ETR/Y) and PY (Y/P) are the tax intensity effect and GDP per capita effect, and P is the population size effect.

As LMDI exhibits excellent theoretical properties, such as simple operation and zero residuals [22], the study used such technique to decompose the change in ETR (ΔETR) over time (year *t* and year *b*), as follows:

(4) $$\Delta ETR=ETR^{t}-ETR^{b}=\Delta TI+\Delta PY+\Delta P$$

By Equation (4), the study can explore the roles of tax intensity (TI), GDP per capita (PY), and population scale (P) in determining changes in ETR at the province level (see Appendix B).

Further, different from most previous studies on ETR focusing on influencing factors over time, the study further depicted the sources of ETR differences among provinces, providing valuable information on reducing unbalanced horizontal distribution of ETR for policymakers. Therefore, following the previous study [14], the difference between the target province *i* and the national average ETR*, Δ*ETR_i_^MR^*, can be expressed by Equation (5):

(5) $$\Delta ETR_{i}^{MR}=ETR_{i}-ETR^{*}$$

Equation (5) can be rewritten as the following formula:

(6) $$\Delta ETR_{i}^{MR}=\sum_{i}TI_{i}\times PY_{i}\times P_{i}=\Delta TR+\Delta PY+\Delta P$$

The difference in province *i* and the regional average ETR can be expressed using the factors in Appendix B. Similarly, Equation (6) implied that regional differences in TI, PY, and P can affect the provincial ETR differences.

### 2.3. Spatial Within-Between LMDI Decomposition Model

Given the driver decomposed above can be also different within- or between-regions, the study further used the spatial within and between LMDI decomposition method to uncover such drivers in accordance with previous research [14]. Therefore, the study designed the target province base within and between regions, which can be rewritten by the following formula:

(7) $$\Delta ETR_{i}^{MR}=ETR_{i}-ETR^{*}\;=\left(ETR_{i}-ETR_{i}^{*}\right)+\left(TER_{i}^{*}-ETR^{*}\right)=\Delta ETR_{i}^{MR-within}+\Delta ETR_{i}^{MR-between}$$

where $ETR_{i}^{*}$ denotes the region *i*’s average ETR, $ETR_{i}^{MR-within}$ denotes the difference between province *i*’s ETR and the regional average ETR, $ETR_{i}^{MR-between}$ denotes the difference between the regional average ETR and the national average level, and $ETR_{i}^{MR-within}$ and $ETR_{i}^{MR-between}$ can be expanded as the followings (see Equations (8) and (9)). The three main drivers of ETR differences between province *i* and the regional average, and the three contributors of ETR differences between the regional average and the national average level, can be expressed by formulas in Appendix B.

(8) $$\Delta ETR_{i}^{MR-within}=\Delta ETR_{i,TI}^{MR-within}+\Delta ETR_{i,PY}^{MR-within}+\Delta ETR_{i,P}^{MR-within}$$

(9) $$\Delta ETR_{i}^{MR-between}=\Delta ETR_{i,TI}^{MR-between}+\Delta ETR_{i,PY}^{MR-between}+\Delta ETR_{i,P}^{MR-between}$$

where $ETR_{i,TI}^{MR-within}$, $ETR_{i,PY}^{MR-within}$, and $ETR_{i,P}^{MR-within}$ denote the within-difference decomposed by *TI*, *PY*, and *P*, and $ETR_{i,TI}^{MR-between}$, $ETR_{i,PY}^{MR-between}$, and $ETR_{i,P}^{MR-between}$ denote the between-difference decomposed by *TI*, *PY*, and *P*. If $ETR_{i,TI}^{MR-within}$ is positive and increases, then the amount of ETR within-difference ($ETR_{i}^{MR-within}$) increases, and vice versa.

### 2.4. Social Network Analysis

SNA is now popularly used in environment or sustainable development issues [17,23]. Here, we employed SNA to depict the spatial pattern of interprovincial ETR networks in China. We first defined the network as a group of nodes connected by links, where “nodes” (here referring to provinces) in the network indicate “participants” [23].

We defined the spatial correlation of ETR as the “line” between two nodes in the network. According to previous studies [18,19], the study used a modified gravity model to build the spatial correlation of interprovincial ETR in the case of China as:

(10) $$y_{ij}=\frac{ETR_{i}}{ETR_{i}+ETR_{j}}\times \frac{\sqrt[{3}]{P_{i}G_{i}ETR_{i}}\times \sqrt[{3}]{P_{j}G_{j}ETR_{i}}}{{\left(D_{ij}/\left(g_{i}-g_{j}\right)\right)}^{2}}$$

where i and j mean compared provinces; $y_{ij}$ is the gravitation of ETR between province i and province j; P, G, g, and D are population, GDP, GDP per capita, and the spherical distance between the provincial capitals, respectively; and $ETR_{i}/\left(ETR_{i}+ETR_{j}\right)$ reflects the gravity coefficient of ETR from province i to province j.

The study can build the gravity matrix of interprovincial ETR according to Equation (10) and obtain the complex interprovincial ETR network above. Then, the network characteristics, including the overall and individual network characteristics, were analyzed (see the previous studies for details) [18,19].

### 2.5. Data

Data on GDP and population over 2000–2019 at the provincial level were collected from the China National Bureau of Statistics, while data on ETR were derived from the China Taxation Yearbook, where GDP and ETR were deflated at constant price in 2000. To be specific, for the price index collected from the China National Bureau of Statistics was in the form of accumulation, starting from 2000.

## 3. Results and Discussions

### 3.1. Changes in Environmental Tax Revenue

**Figure 1:**
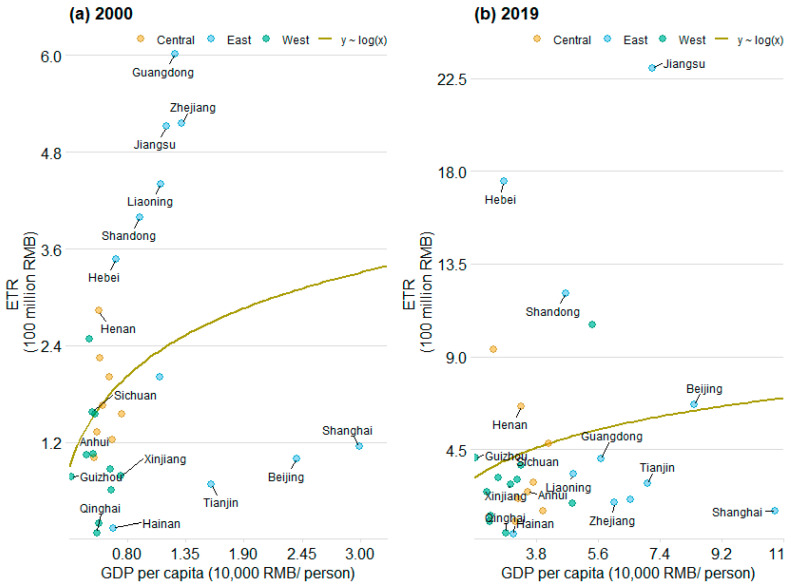
Provincial environmental tax revenue associated with GDP per capita between 2000 and 2019 in China.

Figure 1 indicated the nexus between ETR and economic development over the period. The overall ETR is highly positively correlated with GDP per capita, implying that the higher the economic development level, the higher the ETR. Such a phenomenon exists throughout the sample period, which may be due to that the economic development of China’s provinces, which has not yet achieved full sustainable development [24]. According to the environmental Kuznets curve (EKC) theory [25,26], the pollution level in a certain region will increase first and then decrease with the improvement of economic development. As the object of environmental tax collection is pollution, the results in Figure 1 indicate that China may not have achieved the “turning point” of pollutants at the provincial level. On the other hand, the relative position of ETR and economic development for each province changed during the period. For instance, the larger the slope of the scatter plot line in Figure 1, the higher the ETR, reflecting greater relative pollution under the same GDP per capita. For example, the slope of Jiangsu Province in 2019 is smaller than that in 2005, indicating that Jiangsu Province has made positive achievements in pollution control, which can be also seen in other provinces, such as Zhejiang, Guangdong, Liaoning, and Shanghai.

**Figure 2:**
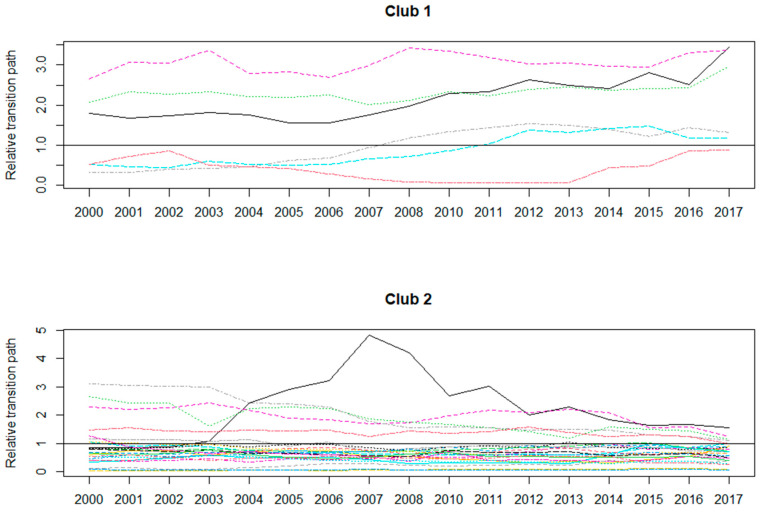
Convergence plots of environmental tax revenue between 2000 and 2017. Note that different colors in Club 1 mean six provinces in the club including Hebei, Jiangsu, Shandong, Inner Mongolia, Jiangxi and Beijing; different colors in Club 2 denote the rest of sample provinces that did not belong to Club 1.

Since China introduced environmental taxes in 2018, which is slightly different from the previous effluent fees, Figure 2 depicts the convergence of ETR at the provincial level between 2000 and 2017. Accordingly, a convergence trend indicates that the variable will be balanced in the long run. Clearly, there are two convergence clubs in terms of China’s ETR, in which club 1 includes Hubei, Jiangsu, Shandong, Inner Mongolia, Jiangxi, and Beijing, while club 2 contains the rest of the 30 sample provinces. This is not consistent with the traditional regional division by the National Bureau of Statistics of China (see Appendix A), where the economic development level of provinces in the eastern region is higher than that in the central region, followed by the western region. Such a result is not similar to Xu et al. (2023) in a case of convergence of fiscal environmental expenditure in China [4], which is mainly due to the different research purposes. Therefore, the convergence results show that future regional collaborative pollution control may also consider convergence-based collaborative policies.

### 3.2. Temporal Drivers of Environmental Tax Revenue

**Figure 3:**
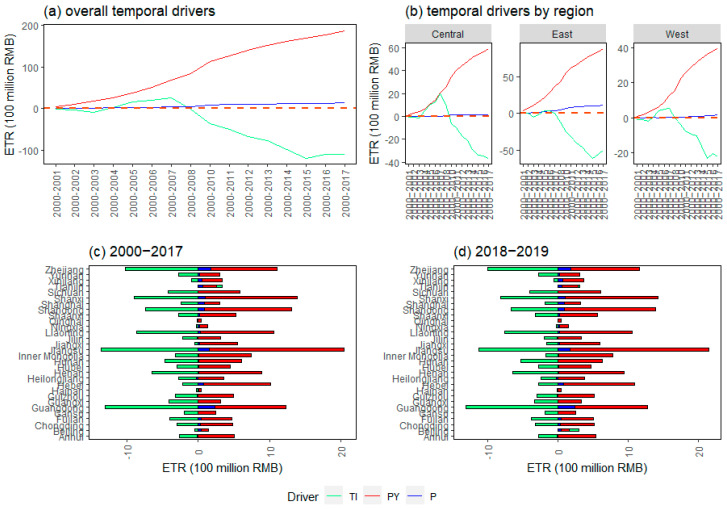
Drivers of environmental tax revenue over time at aggregate levels (**a**,**b**) and provincial levels (**c**,**d**). Note that TI, PY, and P represent tax intensity effect, GDP per capita effect, and population size effect.

Figure 3 reveals the drivers of ETR at the aggregate and regional levels. GDP per capita and population size are the influencing factors of ETR increase in the long term (Figure 3a). However, in comparison with the population size, the effect of GDP per capita on the increase in ETR is dominant. In essence, the level of ETR depends on the level of pollution and the degree of economic development. China has not fully realized the turning point of EKC at the provincial level for a long-term period. That is, the pollution level exhibits trends in first rising and then decreasing with the improvement of economic development. With the continuous economic development, and in the absence of “turning point” of overall pollution, the GDP per capita made a significant impact in promoting the improvement of ETR in the country. In contrast, the population size also plays a certain role in promoting economic development [4]. However, the growth rate of population size is too small in comparison with GDP per capita, resulting in limited impact on the increase in ETR.

As an indicator measuring the “green” degree of environmental tax, the smaller the TI, the smaller the relative pollution. Therefore, Figure 3a shows that the overall TI has an inhibitory effect on the increase in ETR, indicating that TI generally presents a downward trend over the period, which is similar to the finding by Andreoni (2019) to some extent [5]. As mentioned above, the growth rate of ETR is less than that of GDP in the context of the fact that China has not yet reached the “turning point” of pollution during the period. Therefore, the overall TI declined during the sample period.

Figure 3b implies that the conclusions above exhibit certain heterogeneity for different regions. The largest impact of GDP per capita on the absolute change in ETR is the central region over the period. The regions affected by the population size on the absolute change in ETR are in descending order: the eastern, the western, and the central. This is different from the traditional distribution for economic development and reflects the complexity of ETR, which involves both economic development and pollution. TI presents a trend of rising first and then decreasing in the central and western regions, which is consistent with the overall level. In contrast, the impact of TI on the change in ETR in the eastern region was almost always suppressed, which may be due to the smaller relative value of TI in the more developed economy in the eastern region compared with other regions.

Figure 3c illustrated the drivers of the heterogeneity of ETR at the provincial level. There are significant differences in ETR driving factors among provinces even in the same region. For example, although Qinghai and Shaanxi are located in the western region, the role of TI and population size on ETR were almost zero in Qinghai, while the role of TI on ETR was much more significant in Shaanxi. Similar situations also occurred in Anhui and Henan in the central region, as well as Tianjin and Zhejiang in the eastern region. These intraregional driver differences may explain the heterogeneous results of Figure 3b to some extent. Figure 3d depicted the driving factors of ETR at the provincial level since the environmental tax reform. There is no significant change in terms of pattern of each driver, which is consistent with the above findings. A noteworthy point is that, since the relevant tax burden level at the initial stage of levying the environmental tax is basically the same as the previous effluent fees, the analysis is consistent over the whole period.

### 3.3. Spatial Drivers of Environmental Tax Revenue

**Figure 4:**
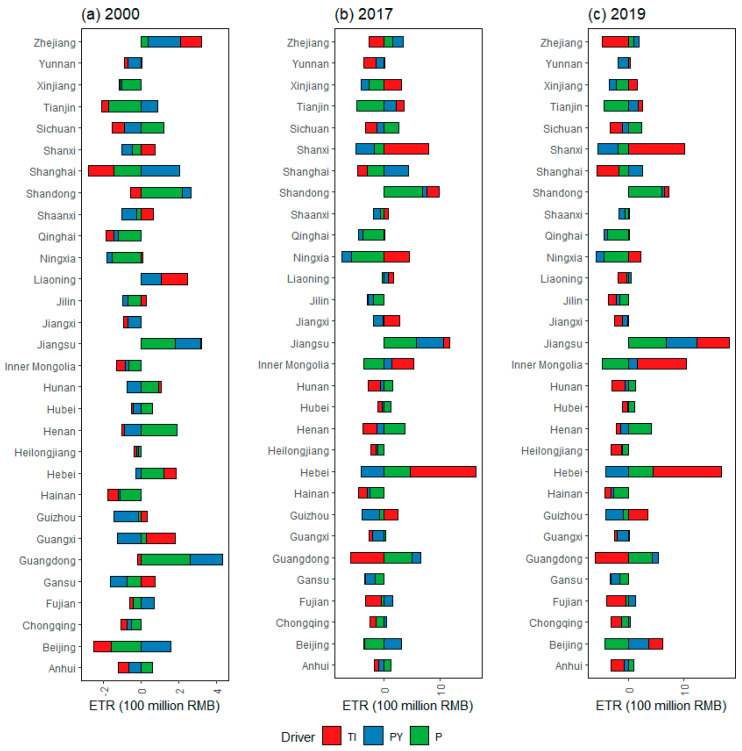
Drivers of difference in environmental tax revenue in representative years under the general spatial LMDI approach. Note that TI, PY, and P represent tax intensity effect, GDP per capita effect, and population size effect.

Figure 4 depicts the drivers of ETR differences at the provincial level, indicating that the provinces whose GDP per capita promotes the ETR gap between the provinces to be evaluated and the benchmark province (the average value of all provincial variables) are Zhejiang, Tianjin, Shanghai, Shandong, Liaoning, Jiangsu, Guangdong, Fujian, and Beijing, respectively. These provinces are generally located in the economically developed regions. For the provinces located in the economically underdeveloped regions in the central and western regions, GDP per capita plays a role in reducing the ETR gap. In Sichuan, Shandong, Jiangsu, Hunan, Henan, Hebei, Guangdong, Anhui, and other provinces, population size plays a role in promoting the expansion of ETR. The impact of TI on the ETR gap also varies from province to province. Interestingly, the effect of population size on the ETR gap is not always less than the GDP per capita, for example, Guangdong and Shandong. This is not consistent with the decomposition results based on the time dimension, which may be due to the different purpose of spatiotemporal decomposition. Time decomposition describes the cause of ETR changes over time, while spatial decomposition represents the cause of ETR difference among provinces. Therefore, the environmental tax policy reform should fully consider the different characteristics across time and space.

In 2017, the driver pattern of interprovincial ETR difference changed significantly compared with 2000. In some provinces, such as Hebei, Shanxi, Shanghai, and Jiangsu, the role of various drivers on the ETR gap has not changed, but the absolute amount of influence has increased, indicating that the importance of ETR in the provinces above has further increased. In contrast, some drivers, such as TI, have changed the direction of influence on the ETR gap in Zhejiang, Tianjin, Shandong, Hunan, Inner Mongolia, Xinjiang, and other provinces, reflecting that these provinces have changed the distribution of TI in all provinces. In comparison with 2017, the impact mode of various drivers on ETR did not change significantly in 2019, implying that the environmental tax was basically the same with the previous effluent fees. Such consistency can contribute to the smooth transition of the policy system after the environmental tax was levied. However, given the unbalanced distribution of ETR and corresponding drivers revealed by the analysis above, a full-fledged system of environmental tax policies at various levels may still be a challenge in the country.

**Figure 5:**
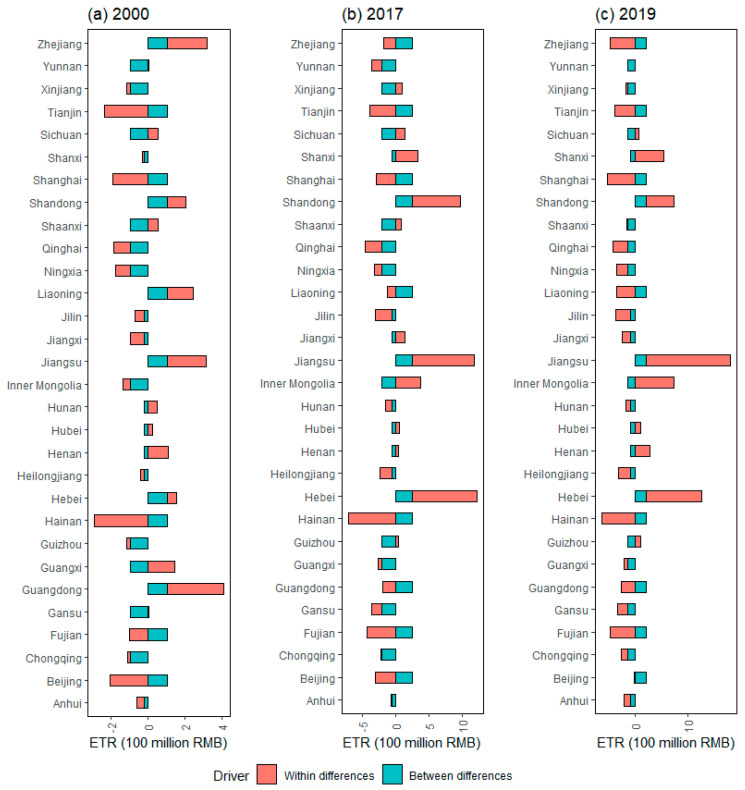
Drivers of difference in environmental tax revenue in representative years under the within and between spatial LMDI approach.

Figure 5 revealed the cause of interprovincial ETR gap from within and between perspectives, and it showed significant regional heterogeneity. The within difference effect is the main cause of the ETR gap for most provinces in terms of the absolute value, including Zhejiang, Tianjin, Shanghai, Liaoning, Jiangsu, Hunan, Henan, Hainan, Guangxi, Guangdong, Beijing, and Anhui. Therefore, in order to balance the overall uneven pattern of ETR distribution, policymakers should focus on narrowing the differences of ETR within the region. However, for different regions, the direction of the effect of within difference is not the same. For example, except for Beijing and Shanghai in the eastern region, the within difference effect plays a promoting role in widening the ETR gap. Beijing and Shanghai are different from other provinces in the eastern region, probably because they have received better policy support and clean energy development, which is more conducive to reducing pollution [19]. For provinces such as Qinghai, Ningxia, Inner Mongolia, and other provinces in the western region, the within difference effect has an inhibitory effect on widening the ETR gap. In 2017 and 2019, the influence direction of within difference effect and between difference effect on ETR gap did not change significantly compared with 2000, which is similar to the findings by the study in a case of CO_2_ emission [14]. To sum up, given that the driver pattern based on within and between perspectives has not changed significantly, the within regional differences should be fully considered in future environmental tax policy reform.

**Figure 6:**
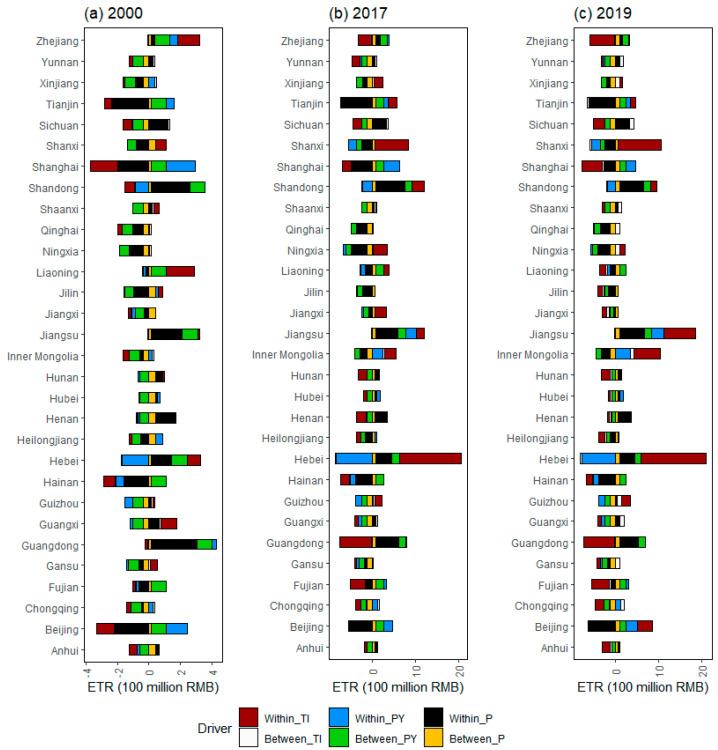
Drivers of difference in environmental tax revenue (ETR) in representative years under the within–between spatial LMDI approach. Note that Within_TI, Within_PY, and Within_P represent intra group effects of tax intensity, GDP per capita, and population size, respectively; Between_TI, Between_PY, and Between_P represent inter group effects of tax intensity, GDP per capita, and population size, respectively.

Figure 6 further revealed what factors within and between regions contributed to the formation of ETR gap by combining the two spatial decomposition perspectives above. In 2000, the within TI difference effect, the between population difference effect, the within GDP per capita difference effect, and the between GDP per capita difference effect were the main causes of the overall ETR gap, while the between population difference effect and the between TI difference effect had very little impact on the ETR gap. Further, the influence of various drivers on the ETR gap is heterogeneous in different regions and within regions. For example, the within TI difference effect suppresses the ETR gap between eastern provinces, such as Shandong, Shanghai, Tianjin, and Beijing, and the benchmark provinces, while it promotes the ETR gap between eastern provinces, such as Zhejiang, Liaoning, Hebei, and the benchmark provinces. In comparison with a previous study [5], such results imply more information on the within and between difference of ETR in the country.

Figure 6 further implied more specific information than the results of Figure 4 and Figure 5. For example, Figure 4 showed that the GDP per capita difference effect is the main promoting factor for the formation of the ETR gap between Zhejiang Province and the benchmark province, while Figure 6 further shows that the GDP per capita difference effect refers to the within GDP per capita difference effect, rather than the between GDP per capita difference effect. Similarly, Figure 5 showed that the within difference effect is the main promoting factor for the formation of the ETR gap between Zhejiang Province and the benchmark province, and Figure 6 further showed that the within difference effect mainly refers to the within GDP per capita difference effect and the within TI difference effect, which is different from a previous study [5]. Therefore, relevant environmental tax policy reform should also fully consider the heterogeneity of variables within and between groups.

### 3.4. Complex Network of Environmental Tax Revenue

**Figure 7:**
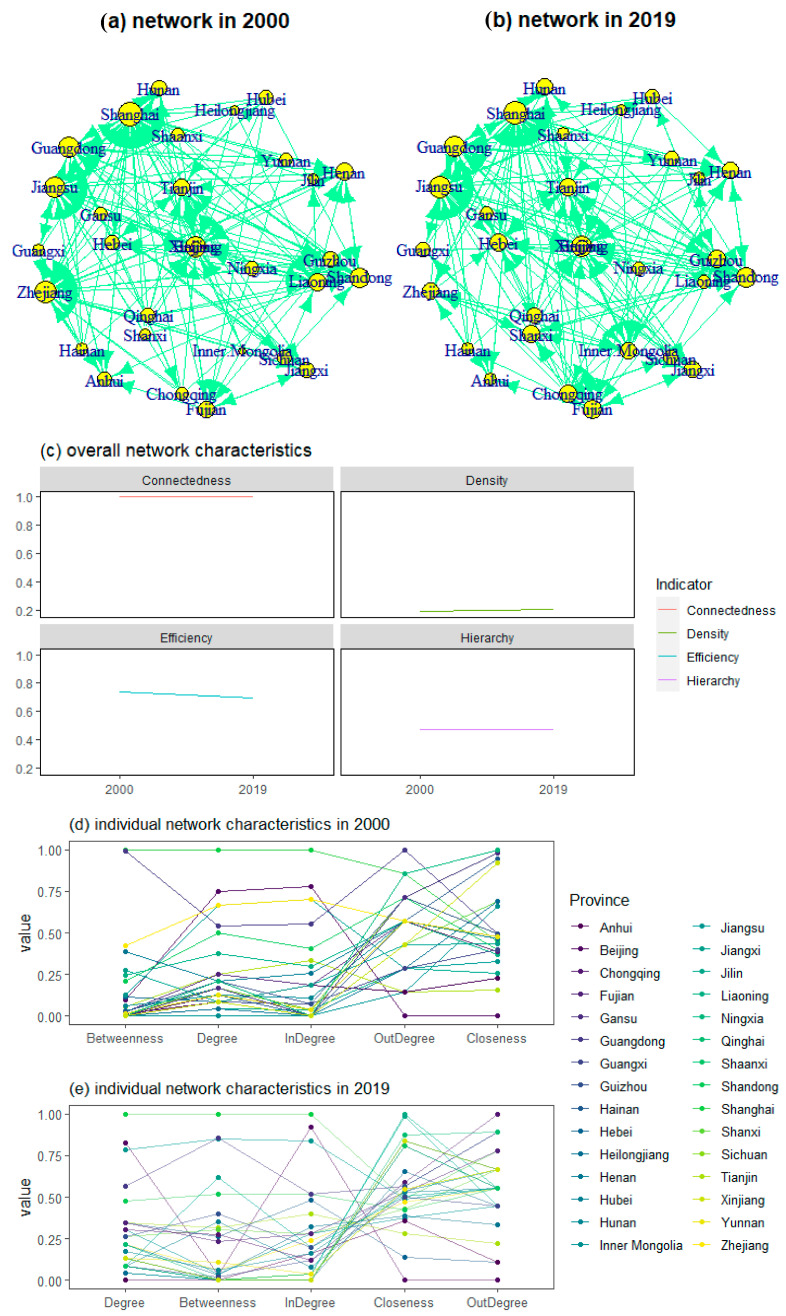
Complex networks of environmental tax revenue (ETR) for China’s provinces between 2000 and 2019.

Figure 7a,b depicted the interprovincial ETR network in China between 2000 and 2019, implying the complex structure of internal connections. In terms of the overall network characteristics, the overall network density of provincial ETR spatial correlation exhibited a slight increasing trend, increasing from 0.1931 in 2000 to 0.2092 in 2019, suggesting the spatial correlation between provinces in China was getting closer in terms of ETR. The overall network connectedness indicated that provincial ETR exhibited significant spillover effects due to the unchanged value of network connectedness. Figure 7c further indicated that the interprovincial ETR network hierarchy presented a slight decreasing trend, changing from 0.4737 in 2000 to 0.4725 in 2019. The finding implied that the interprovincial correlation and interaction of ETR has thus been gradually strengthened after breaking the spatial correlation structure of ETR, which is similar to the study focusing on carbon intensity [19].

The overall network efficiency of the provincial ETR spatial association network (SAN) in China presented a decreasing trend, decreasing from 0.7365 in 2000 to 0.6995 in 2019, indicating that stability has been enhanced after increasing the number of connections in the SAN of ETR. The analysis above implied that the continuous promotion of China’s marketization reform broke the original hierarchical ETR spatial correlation structure, to some extent, which is similar to the existing study [19].

Figure 7d,e depicted the status and role of provinces in SAN of ETR at the individual level. The average degree centrality was 10.07 in 2019, including Beijing, Tianjin, Hebei, Inner Mongolia, Shanghai, Jiangsu, Fujian, Shandong, Guangdong, and Chongqing, whose degree centrality exceeded the average. The provinces above were generally located in the coastal areas, suggesting that the coastal areas have a strong impact on the whole SAN of ETR and spatial spillover effect. Anhui, Yunnan, Guangxi, Jiangxi, Hainan, and Ningxia ranked at the bottom, representing that ETR between the provinces above and other provinces are relatively small. This may be partly due to the relatively small economic scale and remote geographical location of these provinces, resulting in weak spatial correlation in terms of ETR. For the out-degree, the average out-degree centrality of the sample provinces was 6.07 in 2019, where 13 sample provinces did not exceed the average value. The average in-degree centrality was 6.07 in 2019, in which Shanxi, Shanghai, Fujian, and Guangdong were higher than the average of the two degree centralities. In terms of geographical location, most of these provinces are generally located in economically developed coastal areas with massive energy consumption, which is an important source of pollution.

Figure 7e indicated that the average betweenness centrality was 25.83 in 2019, lower than that of Tianjin, Hebei, Shanxi, Shanghai, Jiangsu, Fujian, Jiangxi, Shandong, Hubei, Guangdong, Chongqing, and Guizhou. The provinces above have a high ability to control ETR interactions between other provinces in the SAN of ETR. The average closeness centrality of 30 provinces was 9.56 in 2019, not higher than that of Liaoning, Jilin, Heilongjiang, Guangdong, Hainan, Chongqing, Guizhou, Shaanxi, Gansu, Qinghai, Ningxia, and Xinjiang. The provinces above can connect with other provinces more quickly in the SAN of ETR, which was different from Chen et al. (2022c) on the network pattern of carbon intensity [19]. This may be because these economically less developed provinces were more sensitive for energy usage and ETR.

## 4. Concluding Remarks and Policy Implications

Environmental tax is a special item for prompting economic development and protecting the environment. As an emerging economy, there is still missing knowledge on the in-depth characteristics (e.g., long-term trend, spatiotemporal drivers, and complex network pattern) on the environmental tax revenue in China, hindering further environmental tax policy reforms towards sustainable development. To address the gaps above, this study comprehensively investigated these trends—spatiotemporal drivers and the complex network of provincial environmental tax revenue in the country between 2000 and 2019, by convergence model, as well as spatiotemporal decomposition models and social network analysis models, respectively. The key findings are listed as follows.

First, two convergence clubs of ETR for China’s provinces over the period were found. Second, GDP per capita and tax intensity were positive and negative drivers, contributing to the increase in ETR. Third, within differences in tax intensity and GDP per capita, and the between differences in population and GDP per capita, were the main driving factors widening the overall ETR gap. Fourth, the original hierarchical ETR spatial correlation structure has changed, while sample provinces exhibited certain degrees of heterogeneity in terms of SAN of ETR.

We provide the following policy recommendations based on the results above.

First, we suggest that relevant departments can formulate differentiated environmental tax policies according to regional development characteristics. The study indicated that the impact of the influencing factors on the environmental tax revenue in different regions was heterogeneous, which was highly related to the degree of economic development in each region. Therefore, one efficient way towards protecting the environment and promoting economic development by environmental tax revenue is to constantly develop the economy by local conditions, such as resource endowment and development orientation according to comparative advantage theory [27]. In addition, the decision-makers should also consider reducing the tax burden of other taxes, such as value-added tax and enterprise income tax, so that the overall tax burden can be reduced or unchanged and the cost can be reduced. Such implication can contribute to effectively promoting regional economic development while protecting the environment.

Second, we suggest that the relevant departments can formulate a coordinated regional environmental tax policy to promote economic development and protect the environment, simultaneously. The results of the study implied that there were convergence clubs for environmental tax revenue among different regions. The regional coordination policy of environmental tax based on convergence, thus, may also be a feasible choice. This is different from the previous regional policies based on geographical or economic characteristics (e.g., Beijing-Tianjin-Hebei urban agglomeration, as well as the Pearl River Delta and the Yangtze River Delta). The regional coordination policy based on convergence considers the equilibrium in the long run and may be a helpful supplement to the previous regional coordination policy. On the other hand, this study also suggested that the environmental tax revenue has a spatial correlation effect, and corresponding complex network structure was more closely connected over the period. Therefore, the regional network correlation characteristics should also be fully considered when formulating relevant policies.

Third, we suggest that relevant policy makers can establish a tracking system of environmental tax revenue. The system can include the functions of tracking long-term trends, identifying driving factors, as well as mode judgments of related networks, etc. Meanwhile, the relevant departments should also update the relevant data in a timely manner in order to maximize the policy reference value of the tracking system. With respect to this, policy makers can adjust the environmental tax policy more scientifically and timely.

## 5. Limitations and Research Prospect

The study has some limitations. First, this study did not conduct analysis over a longer period and at smaller research scale (e.g., city level) due to the limitation of data. Second, although the study employed the newly developed spatial within and between decomposition approach and obtained some new findings, some relationships between ETR and other socioeconomic variables, such as decoupling and Granger causality relationships, are lacking in discussion. Those limitations will be addressed in future studies. In addition, future research can be also conducted by combining tools such as difference-in-difference (DID) to discussion the impact of specific environmental tax policy on corresponding tax revenue.

## Data Availability

Not applicable.

## Appendix A

**Table A1:** Classifications on China’s provinces according to the National Bureau of Statistics of China.

Standard	Region	Province
Normal	North	Beijing, Tianjin, Hebei, Shanxi, Inner Mongolia
	Northeast	Liaoning, Jilin, Heilongjiang
	East	Shanghai, Jiangsu, Zhejiang, Anhui, Fujian, Jiangxi, Shandong
	Central south	Henan, Hubei, Hunan, Guangdong, Guangxi, Hainan
	Southwest	Chongqing, Sichuan, Guizhou, Yunnan, Xizang
	Northwest	Shaanxi, Gansu, Qinghai, Ningxia, Xinjiang
Hot	Yangtze River Delta	Shanghai, Jiangsu, Zhejiang
	Bohai Rim region	Beijing, Tianjin, Hebei, Liaoning, Shandong
	Pan Pearl River Delta	Fujian, Jiangxi, Hunan, Guangdong, Guangxi, Hainan, Sichuan, Guizhou, Yunnan
Eight	Northeast	Liaoning, Jilin, Heilongjiang
	North coast	Beijing, Tianjin, Hebei, Shandong
	East coast	Shanghai, Jiangsu, Zhejiang
	South coast	Fujian, Guangdong, Hainan
	Middle reaches of the Yellow River	Shanxi, Inner Mongolia, Henan, Shaanxi
	Middle reaches of the Yangtze River	Anhui, Jiangxi, Hubei, Hunan
	Southwest	Guangxi, Chongqing, Sichuan, Guizhou, Yunnan
	Northwest	Xizang, Gansu, Qinghai, Ningxia, Xinjiang
Three	Eastern	Beijing, Tianjin, Hebei, Liaoning, Shanghai, Jiangsu, Zhejiang, Fujian, Shandong, Guangdong, Hainan
	Central	Shanxi, Jilin, Heilongjiang, Anhui, Jiangxi, Henan, Hubei, Hunan
	Western	Inner Mongolia, Guangxi, Chongqing, Sichuan, Guizhou, Yunnan, Xizang, Shaanxi, Gansu, Qinghai, Ningxia, Xinjiang

## Appendix B

The decompositions on Equation (4) are listed as follows:

$$\Delta TR=\sum_{i}L\left(ETR^{t},ETR^{b}\right)\times \ln\left(\frac{TI_{i}^{t}}{TI_{i}^{b}}\right)\;\Delta PY=\sum_{i}L\left(ETR^{t},ETR^{b}\right)\times \ln\left(\frac{PY_{i}^{t}}{PY_{i}^{b}}\right)\;\Delta P=\sum_{i}L\left(ETR^{t},ETR^{b}\right)\times \ln\left(\frac{P_{i}^{t}}{P_{i}^{b}}\right)$$

where $L\left(ETR_{i}^{t},ETR_{i}^{b}\right)=\left(ETR_{i}^{t},ETR_{i}^{b}\right)/\left(\ln ETR_{i}^{t}-\ln ETR_{i}^{b}\right)$ is the logarithmic mean weight of LDMI decomposition.

The decompositions on Equation (4) are listed as follows:

$$\Delta ETR_{i,TI}^{MR}=\sum_{i}L\left(ETR_{i},ETR^{*}\right)\cdot \ln\left(TI_{i}/TI^{*}\right)\;\Delta ETR_{i,P}^{MR}=\sum_{i}L\left(ETR_{i},ETR^{*}\right)\cdot \ln\left(P_{i}/P^{*}\right)\;\Delta ETR_{i,PY}^{MR}=\sum_{i}L\left(ETR_{i},ETR^{*}\right)\cdot \ln\left(PY_{i}/PY^{*}\right)$$

where $L\left(ETR_{i},ETR^{*}\right)=\left(ETR_{i}-ETR^{*}\right)/\left(\ln ETR_{i}-\ln ETR^{*}\right)$ is the spatial logarithmic mean weight.

The decompositions on Equations (8) and (9) are listed as follows:

$$\Delta ETR_{i,TI}^{MR-within}=\sum_{i}L\left(ETR_{i},ETR_{i}^{*}\right)\cdot \ln\left(TI_{i}/TI^{*}\right)\;\Delta ETR_{i,SC}^{MR-within}=\sum_{i}L\left(ETR_{i},ETR_{i}^{*}\right)\cdot \ln\left(PY_{i}/PY^{*}\right)\;\Delta ETR_{i,G}^{MR-within}=\sum_{i}L\left(ETR_{i},ETR_{i}^{*}\right)\cdot \ln\left(P_{i}/P^{*}\right)\;\Delta ETR_{i,TI}^{MR-between}=\sum_{i}L\left(ETR_{i}^{*},ETR^{*}\right)\cdot \ln\left(TI_{i}^{*}/TI^{*}\right)\;\Delta ETR_{i,SC}^{MR-between}=\sum_{i}L\left(ETR_{i}^{*},ETR^{*}\right)\cdot \ln\left(PY_{i}^{*}/PY^{*}\right)\;\Delta ETR_{i,G}^{MR-between}=\sum_{i}L\left(ETR_{i}^{*},ETR^{*}\right)\cdot \ln\left(P_{i}^{*}/P^{*}\right)$$

where $L\left(ETR_{i},ETR_{i}^{*}\right)=\left(ETR_{i}-ETR_{i}^{*}\right)/\left(\ln ETR_{i}-\ln ETR_{i}^{*}\right)$ and $L\left(ETR_{i}^{*},ETR^{*}\right)=\left(ETR_{i}^{*}-ETR^{*}\right)/\left(\ln ETR_{i}^{*}-\ln ETR^{*}\right)$ mean the logarithmic mean weights of spatial LMDI within the decomposition and the between decomposition, respectively.
